# Sinomenine Confers Protection Against Myocardial Ischemia Reperfusion Injury by Preventing Oxidative Stress, Cellular Apoptosis, and Inflammation

**DOI:** 10.3389/fphar.2022.922484

**Published:** 2022-06-28

**Authors:** Boyu Xia, Qi Li, Jingjing Wu, Xiaomei Yuan, Fei Wang, Xu Lu, Chao Huang, Koulong Zheng, Rongrong Yang, Le Yin, Kun Liu, Qingsheng You

**Affiliations:** ^1^ Department of Cardiothoracic Surgery, Affiliated Hospital of Nantong University, Nantong, China; ^2^ Department of Cardiology, Suzhou Kowloon Hospital of Shanghai Jiaotong University School of Medicine, Suzhou, China; ^3^ Department of Cardiology, Sichuan Provincial People’s Hospital, University of Electronic Science and Technology of China, Chengdu, China; ^4^ Department of Pharmacology, School of Pharmacy, Nantong University, Nantong, China; ^5^ Department of Cardiology, The Second Affiliated Hospital of Nantong University, Nantong, China; ^6^ Department of Anesthesiology, Affiliated Hospital of Nantong University, Nantong, China; ^7^ Department of Cardiology, Tongzhou People’s Hospital, Nantong, China

**Keywords:** sinomenine, ischemia reperfusion injury, oxidative stress, inflammation, apoptosis

## Abstract

Sinomenine (SIN), an alkaloid extracted from the root of *S. acutum. sinomenine*, has been shown to have antiarrhythmic, antioxidant, and anti-inflammatory effects in myocardial ischemia-reperfusion injury (MIRI) *ex vivo*. In this study, we investigated the cardioprotective effects of SIN in an *in vivo* mouse model of MIRI. Adult male C57BL/6J mice received SIN (80 mg/kg) for 5 days and underwent 30 min of percutaneous occlusion of the left anterior descending artery (LAD) followed by 24 h of reperfusion. Results showed that pretreatment with SIN significantly reduced myocardial infarct size and concentrations of markers of cardiac injury and improved left ventricular ejection fraction (EF) and shortening fraction (FS) in MIRI mice. The SIN pretreatment prevented the MIRI-induced decrease in the expression levels of Bcl-2, increase in the expression levels of caspase-3, caspase-9, and Bax, and increase in the number of TUNEL-positive cells in ischemic heart tissue. It was also found that pretreatment with SIN prevented the MIRI-induced oxidative stress imbalance in ischemic heart tissue, as shown by the increase in total antioxidant capacity (T-AOC) and glutathione (GSH) and the decrease in malondialdehyde (MDA), reactive oxygen species (ROS), and dihydroethidium (DHE) density. Further studies showed that the stimulus of cardiac ischemia/reperfusion caused a remarkable increase in the expression levels of interleukin-1β (IL-1β), IL-6, and tumor necrosis factor-α (TNF-α) mRNA in ischemic heart tissue, which was effectively prevented by pretreatment with SIN. These results demonstrate that SIN can attenuate MIRI-induced cardiac injury *in vivo* by preventing oxidative stress, inflammation, and apoptosis.

## Introduction

Coronary artery disease (CAD) remains the leading cause of death in the 21st century, accounting for 16% of all deaths worldwide (World Health Organization, 2020). Pathological studies show that plaque in the vessels can narrow the vascular cavity and eventually block the flow of oxygenated blood to the heart. Timely unblocking of occluded arteries, known as reperfusion, can significantly reduce mortality and morbidity (Hanson et al., 2013). However, restoration of blood flow to ischemic regions may also cause additional damage to cardiac tissue, termed myocardial ischemia-reperfusion injury (MIRI) (Carden and Granger, 2000). Although advanced technologies and newly discovered antiplatelet and antithrombotic agents have been shown to be effective in improving MIRI, there are no standard strategies for the prevention and/or treatment of MIRI (Heusch, 2020). The search for new drugs for MIRI prevention and treatment is of great importance in the clinic.

Oxidative stress has been shown to mediate the process of acute ischemia-reperfusion injury (Kalogeris et al., 2012). Here, we focused on the multiple mechanisms that include reactive oxygen species (ROS) production, inflammation, and cellular apoptosis (Hausenloy and Yellon, 2013; Ashraf et al., 2014; Granger and Kvietys, 2015; Kalogeris et al., 2016; Jin et al., 2017; Zhai et al., 2017). Myocardial necrosis results from the initial ischemic insult, and further damage caused by reperfusion is due to metabolic changes in oxygen recovery by ischemic mitochondria (Lesnefsky et al., 2017). The progression of reperfusion can lead to excessive accumulation of ROS, which subsequently act as inflammatory signaling mediators and trigger an inflammatory response (Lesnefsky et al., 2017). The inflammatory response and oxidative stress reinforce each other and eventually lead to programmed cell death of the myocardium (Lesnefsky et al., 2017).

Sinomenine (SIN), an alkaloid extracted from the root of the Chinese medicinal plant *S. acutum. sinomenine,* has been reported to have potent pharmacological effects, ranging from anti-inflammatory, analgesic, immunosuppressive, antioxidant, anti-apoptotic, and anti-cancer (Qian et al., 2007; Lu et al., 2013; Zhou et al., 2017; Gao et al., 2019). Some previous studies have shown the successful use of SIN in the treatment of rheumatoid arthritis and other inflammatory diseases in China and Japan (Zhao et al., 2012; Tang et al., 2021). Mechanistic studies have reported that SIN achieves the above pharmacological effects mainly by suppressing p38 mitogen-activated protein kinases (MAPK)/nuclear factor-κB (NF-κB) signaling (Shen et al., 2018), restoring the balance of oxidative stress in an NF-E2-related factor 2 (Nrf2)-dependent manner (Qin et al., 2016; Zhang et al., 2019), or reducing ROS production (Shukla and Sharma, 2011; Fan et al., 2017). Administration of SIN can also trigger protective functions in the cardiovascular system (Xie and Jin, 1993; Nishida and Satoh, 2007a). For example, treatment with SIN has been shown to improve stress-induced heart failure by inhibiting the expression of collagen type I and III and increasing the ratio of interleukin-10 (IL-10)/IL-17 (Fu et al., 2020). When combined with cyclosporine A, SIN effectively inhibits angiogenesis and tissue remodeling in chronic heart transplant rejection (Mark et al., 2003). In addition, administration of SIN has been shown to maintain cardiovascular functions in hypertension or norepinephrine-induced vasoconstriction by controlling cardiac ion channels and mediating endothelium-dependent vasodilation (Wallukat, 2002; Nishida and Satoh, 2007b; Lee et al., 2007).

Although previous studies have confirmed the anti-oxidative stress and anti-inflammatory properties of SIN in MIRI *ex vivo* (Xie and Jin, 1993; Lu et al., 2022), the *in vivo* function of SIN in MIRI has not yet been elucidated. We propose that administration of SIN may be able to prevent ischemia/reperfusion-induced cardiac injury by preventing oxidative stress, cellular apoptosis, and inflammation *in vivo*.

## Materials and Methods

### Materials

The SIN (#HY-15122, MCE, New Jersey, United States) was dissolved in 10% DMSO with 40% PEG300, 5% Tween-80, and 45% saline. Solutions containing only 10% DMSO, 40% PEG300, 5% Tween-80, and 45% saline were used as vehicles.

### Animals

Adult male C57BL/6J mice (6–8 weeks old, Beijing Vital River Laboratory Animal Technology, China) were housed five per cage in standard vivarium conditions (22°C, 55% relative humidity, and 12 h light/darkness) with *ad libitum* access to water and food. The animal experiments were approved by the Animal Ethics Committee of Nantong University (Permit Number: 2110836) and were conducted in accordance with the internationally accepted guidelines for the use of animals in toxicology adopted by the Society of Toxicology in 1999.

### Experimental Design and Drug Treatment

**FIGURE 1:**
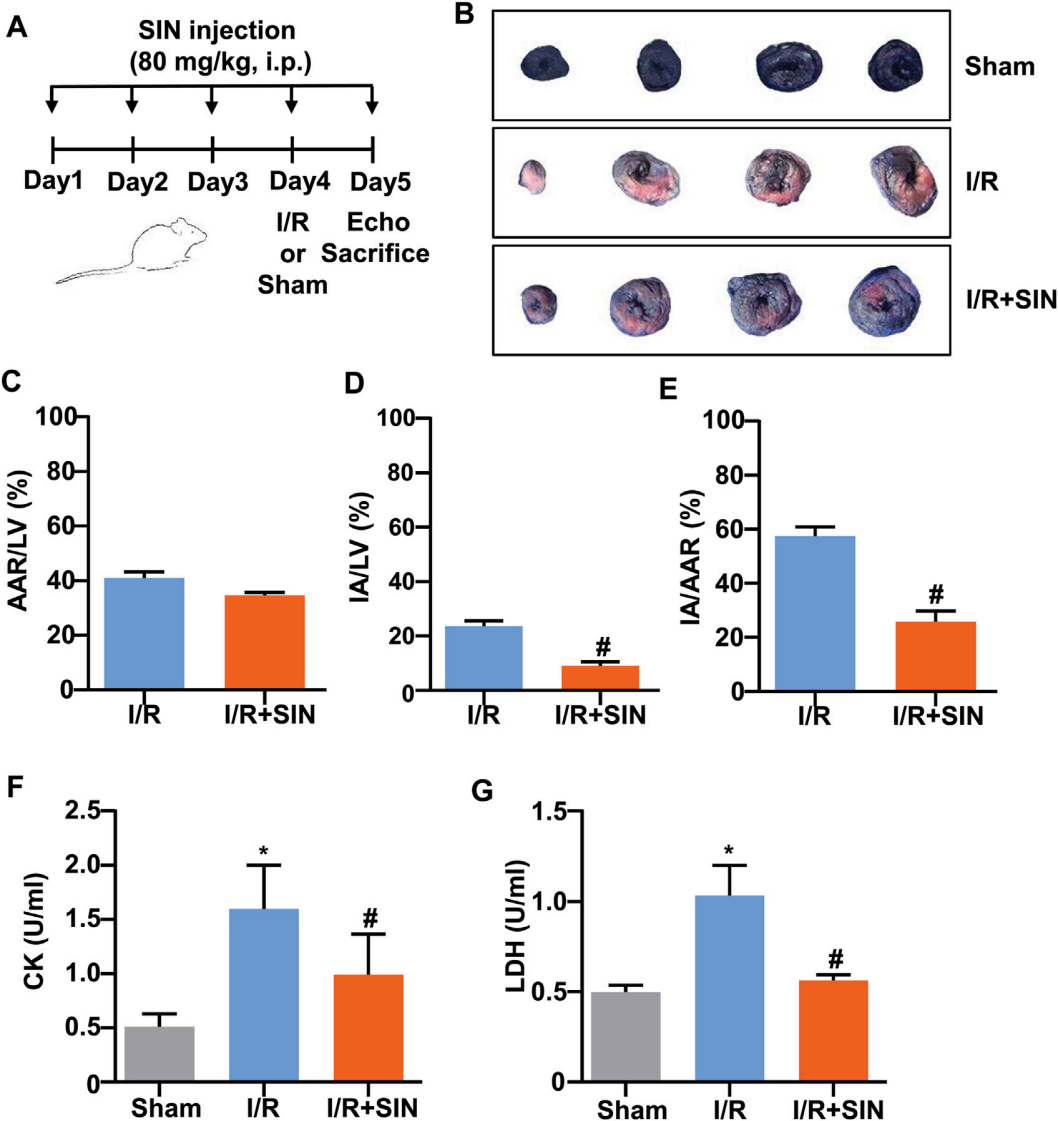
Effect of SIN on myocardial damage in MIRI mice. **(A)** Timeline for the experimental showing drug administration, ischemia-reperfusion surgery, and killing of the mice. **(B)** Representative photograph of Evans blue/TTC double-staining in the heart of mice treated with or without SIN. The non-ischemic area, which was not at risk, was stained blue, whereas the viable myocardium was stained red and the infarct area was stained white. The area at risk was the sum of the white and red regions. **(C–E)** Quantitative analysis of Evans blue/TTC double staining show that pretreatment with SIN significantly decreased the ratio of infarct area to left ventricle (IA/LV) (C, *n* = 5) and area at risk (IA/AAR) (E, *n* = 5) compared with the MIRI group (#*p* < 0.05 vs. I/R), and no significant differences in area at risk normalized to left ventricle (AAR/LV) (D, *n* = 5) were observed between the two groups. **(F,G)** Quantitative analysis show that pretreatment with SIN significantly prevented the MIRI-induced increase in serum CK (F, *n* = 5) and LDH (G, *n* = 5) levels in mice (*p < 0.05 vs. sham; ^#^ *p* < 0.05 vs. I/R). Data are shown as means ± SEM.

After 1 week of acclimatization, 24 mice were randomly divided into sham, I/R, and I/R + SIN groups (*n* = 8 in each group). SIN was administered to the animals by intraperitoneal injection at a dose of 80 mg/kg once daily for five consecutive days before surgery (Gao et al., 2013; Ma et al., 2016). The myocardial ischemia/reperfusion (I/R) model was established on day 4. After reperfusion, mice underwent echocardiography and then were sacrificed for further analysis (Figure 1A). Samples of cardiac tissue were obtained from the ischemic area of the left ventricle.

### Establishment of the Myocardial I/R Model

The procedures for myocardial I/R were described below (Gao et al., 2010). Mice were anesthetized with inhaled isoflurane via an isoflurane delivery system (Yuyan Instruments, Shanghai, China). Then, a small incision was made in the left chest, followed by blunt dissection of the subcutaneous tissue. After that, the pleura was punctured with eye forceps through the fourth intercostal space. Then, the heart was removed manually by pressing on the upper back of the mouse. Ischemia was induced with a slip knot around the left anterior descending artery (LAD) approximately 2 mm from its origin with a 6–0 polyamide 6 suture. Effective ligation was confirmed by the appearance of a pale color in the anterior wall of the left ventricle. Thirty minutes later, the slipknot was released to reperfuse the LAD for 24 h. The sham-operated group underwent a similar procedure, except for closure of LAD. The animals received subcutaneous buprenorphine (0.05 mg/kg) for postoperative analgesia (Morel et al., 2014). The survival rate after surgery was approximately 80%.

### Determination of Myocardial Infarct Size

Double staining with Evans Blue and TTC (2, 3, 5-triphenyltetrazolium chloride) was performed to determine infarct size (Pu et al., 2013). Briefly, the LAD was ligated immediately after 24 h of reperfusion, and then 1.5 ml of 1% Evans Blue dye was injected into the left ventricle. Then, the heart was rapidly excised, frozen at −20°C, and cut into 2-mm-thick sections perpendicular to the long axis, resulting in four sections per heart. The tissues sections were then incubated in a 1% TTC solution at 37°C for 20 min, and the tissue images were captured using a Canon 600D digital single-lens reflex (DSLR) camera. In this experiment, the blue regions indicated normal myocardium, the red regions represented ischemic but not infarct-prone areas, and the pale white area was the infarct area (IA). The area at risk (AAR) was composed of white and red regions. Staining was evaluated with Image-Pro Plus 6.0 (Media Cybernetics, Torrance, CA, United States), with results taken as AAR/LV, IA/LV, and IA/AAR × 100%.

### Measurements of CK and LDH Levels

To obtain serum samples, blood was drawn and centrifuged at 1,000 g for 10 min at 4°C to obtain serum samples. Subsequently, serum levels of CK and LDH were determined using commercial detection kits (Nanjing Jiancheng Bioengineering Institute, Nanjing, China) according to the manufacturer’s instructions (Qu et al., 2016).

### Echocardiographic Analysis of Cardiac Function

At the end of reperfusion, mice were anesthetized with isoflurane and examined with an *in vivo* ultrasound system for small animal models (VEVO 2100, Visual Sonics, Toronto, Canada) (Pu et al., 2013). M-mode recordings were recorded in a short-axis view of the left ventricle at the level of the papillary muscle. The inner diameter of the left ventricle at the end of diastole (LVIDd) and at the end of systole (LVIDs) was then measured in the recordings. Left ventricular ejection fraction (EF) and fractional shortening (FS) were calculated according to the following formula: EF = [(LVIDd)^3^−(LVIDs)^3^]/(LVIDd)^3^ × 100%; FS = (LVIDd−LVIDs)/LVIDd × 100%, respectively. Measurements were averaged from three consecutive cardiac cycles and were performed by an experienced technician who was blinded to group assignment.

### TUNEL Assay

The TUNEL assay was performed using the One-Step TUNEL Apoptosis Assay Kit (Beyotime, Shanghai, China) (Meng et al., 2021). For this experiment, the left ventricles of mice were harvested, fixed with 4% paraformaldehyde (PFA), dehydrated with sucrose, embedded in OCT, and cut into 8-μm-thick sections. The tissue sections were treated with PFA, permeabilized with 0.5% Triton X-100, and incubated in working buffer at 37°C for 60 min according to the manufacturer’s instructions. Then the slides were sealed with glycerol and analyzed under a fluorescence microscope (Leica, Wetzlar, Germany).

### Measurements of Malondialdehyde, Glutathione, and Total Antioxidant Capacity Concentrations

MDA, GSH, and T-AOC concentrations were measured in tissue homogenates of the left ventricle using commercial kits (Nanjing Jiancheng Bioengineering Institute, Nanjing, China) and analyzed by the spectrophotometric quantification method (Qu et al., 2016). Results were normalized against total protein using the BCA protein assay (Beyotime, Shanghai, China) and expressed as nmol of MDA, nmol of GSH, and units of T-AOC per mg of protein in the homogenate.

### Isolation of Mitochondria

Mitochondria were isolated from equilibrated left ventricular tissue using the Mitochondrial Isolation Kit (Beyotime, Shanghai, China) according to the manufacturer’s instructions (Yang et al., 2017). In brief, mice were euthanized and their left ventricles were rapidly excised and transferred to ice-cold PBS. The ischemic region distal to the original suture ligation was crushed and trypsinized in an ice bath. It was then homogenized with a Potter-Elvehjem tissue grinder in the reagent A for isolation of mitochondria included in the kit and centrifuged at 600 g for 5 min at 4°C. Mitochondria were recovered by further centrifugation of the supernatant at 11,000 g for 10 min and suspended in 40 μl of the mitochondria storage fluid from the kit.

### Reactive Oxygen Species Assay

The mitochondrial ROS assay was performed using the ROS assay kit (Beyotime, Shanghai, China), containing 20,70-dichlorofluorescein diacetate (DCFH-DA), a substance that can be cleaved to DCFH in the presence of H_2_O_2_, to monitor the level of ROS (Lin et al., 2021). The fluorescence intensity of DCFH was determined using the microplate reader (Synergy H1, BioTek, United States) at an excitation wavelength of 488 nm and an emission wavelength of 525 nm. The results were expressed as a percentage of the sham.

### Dihydroethidium Staining

Dihydroethidium (DHE) staining was performed according to the manufacturer’s protocols (Liu et al., 2020). In brief, 8 μm-thick frozen left ventricular sections were incubated with DHE solution (Servicebio, Wuhan, China) for 30 min at 37°C, and then the sections were viewed under a fluorescence microscope (Leica, Wetzlar, Germany).

### RNA Extraction and Real-Time PCR

According to the manufacturer’s instructions, total RNA was extracted from left ventricular tissues of mice treated with or without cardiac I/R injury and SIN using the RNAeasy total RNA extraction kit (Qiagen, GmbH, Hilden, Germany). The cDNA was generated with the reverse transcription kit from Promega (Madison, WI, United States). Glyceraldehyde-3-phosphate dehydrogenase (GAPDH) was used as an internal control. Real-time PCR was performed using a reaction system containing 1 × Faststart SYBR Green Master Mix (Roche Molecular Biochemicals), 2 μl diluted cDNA, 2 mM MgCl_2_, and 0.5 μM primer. The experiment was repeated three times. Primers used in the experiments were cited as follows: TNF-α, 5′-CTG​TGA​AGG​GAA​TGG​GTG​TT-3′ (F), 5′-GGTCAC TGTCCCAGCATCTT-3′ (R); IL-β, 5′-TGGAAAAGC GGTTTGTC TTC-3′ (F), 5′-TAC​CAG​TTG​GGG​AAC​TCT​GC-3′ (R); IL-6: 5′-AGA​GAT​ACA​AAG​AAA​TGA​TGG​A-3′ (F), 5′-AGC​TAT​GGT​ACT​CCA​CAA-GAC​CA-3′ (R); GAPDH: 5′-GGC​CTT​CCG​TGT​TCC​TAC-3′ (F), 5′-TGT​CAT​CAT​ATC​TGG​CAG​GTT-3′ (R) (Huang et al., 2018).

### Western Blot

Total proteins were extracted from left ventricular tissues of mice treated with or without cardiac I/R injury and SIN, and protein concentrations were determined using a BCA protein assay (Beyotime, Shanghai, China) (Su et al., 2022). Samples were boiled with loading buffer at 95°C for 10 min, separated at 80 V for 1.5 h on 10% SDS-PAGE gels, and then transferred to nitrocellulose membranes (Merck Millipore, Darmstadt, Germany) at 260 mA for 90 min. After blocking with nonfat milk for 2 h at room temperature, the nitrocellulose membranes were treated overnight at 4°C with antibodies against caspase 3 (1:500; 19677-1-AP, Proteintech), caspase 9 (1:500; 10380-1-AP, Proteintech), Bax (1:500; 60267-1-Ig, Proteintech), Bcl-2 (1:500; 60178-1-Ig, Proteintech), and GAPDH (1:8000; 10494-1-AP, Proteintech). The primary antibodies were then removed by washing the membranes three times in Tris-buffered saline and Tween 20 (TBST) and incubated for an additional 2 h at room temperature with IRDye 680-labeled secondary antibodies (1:10000; 926-68072, 926-68073, LI-COR). Immunoblot bands were visualized by scanning with the Odyssey CLx Western blot detection system (LICOR, Nebraska, United States), and band density was quantified using ImageJ software (NIH, United States).

### Statistics

All data are expressed as mean ± standard error of the mean (SEM) and were analyzed by unpaired Student’s t test or one-way ANOVA with repeated measures followed by Bonferroni multiple comparisons on GraphPad Prism 8 (Graphpad Software, California, United States). A value of *p* < 0.05 was considered statistically significant.

## Results

### Sinomenine Prevented Myocardial Injury Induced by Myocardial Ischemia-Reperfusion Injury in Mice

**FIGURE 2:**
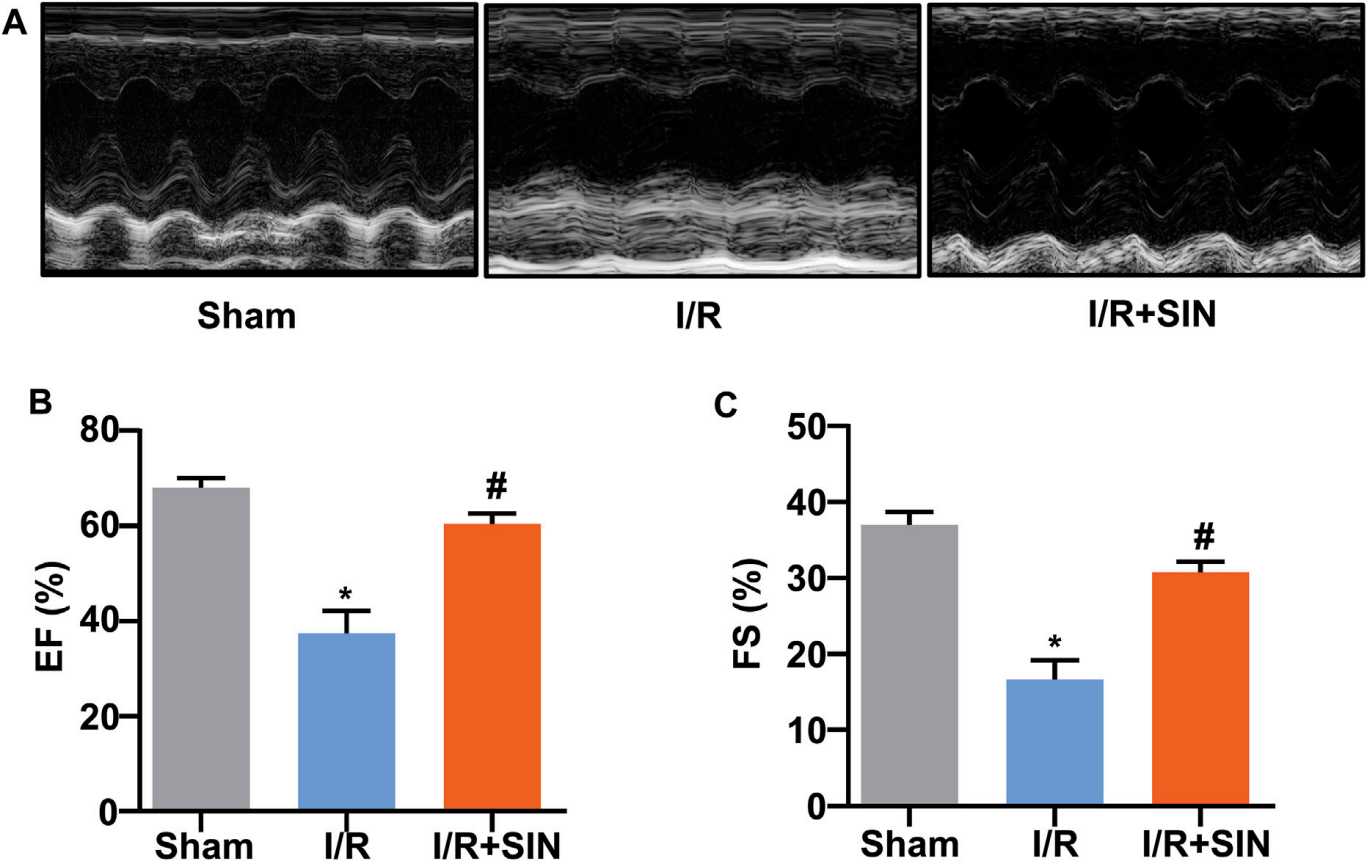
Effect of SIN on cardiac structure and function in MIRI mice. **(A)** Representative images show the changes in cardiac structure detected by echocardiography in mice treated with or without MIRI and/or SIN. **(B,C)** Quantitative analysis show that pretreatment with SIN prevented the MIRI-induced decrease in ejection fraction EF, **(B)** and fractional shortening FS, **(C)** values (*n* = 5, *p < 0.05 vs. sham, ^#^ *p* < 0.05 vs. I/R). Data are shown as means ± SEM.

As shown in Figures 1B‒E, no significant difference in the ratio of area at risk to left ventricle (AAR/LV) was observed between the I/R and I/R + SIN groups. However, compared with the I/R mice, the ratio of infarct area to area at risk (IA/AAR) was reduced from 57.53 ± 1.48% to 25.90 ± 1.73% (*t*_*8*_ = 13.89, *p* < 0.001) and the ratio of infarct area to left ventricle (IA/LV) was reduced from 23.57 ± 0.90% to 9.01 ± 0.69% (*t*_*8*_ = 12.84, *p* < 0.001) in the SIN-treated mice. The SIN pretreatment also prevented the concomitant increase in serum levels of CK (1.60 ± 0.18 in I/R vs. 0.99 ± 0.17 in I/R + SIN; F_2, 12_ = 13.93, *p* < 0.001; Figure 1F) and LDH (1.03 ± 0.08 in I/R vs. 0.56 ± 0.02 in I/R + SIN; F_2, 12_ = 41.34, *p* < 0.001; Figure 1G) in mice receiving cardiac I/R, suggesting that SIN is able to attenuate myocardial necrosis induced by cardiac I/R. Subsequently, echocardiography was used to investigate the protective effect of SIN on cardiac functions 24 h after I/R (Figure 2A). We found that pretreatment with SIN significantly prevented the I/R-induced decrease in the levels of EF (37.36 ± 2.09% with I/R vs. 60.43 ± 0.96% with I/R + SIN; F_2, 12_ = 122.6, *p* < 0.001; Figures 2A,B) and FS (16.63 ± 1.14% with I/R vs. 30.77 ± 0.63% with I/R + SIN; F_2, 12_ = 141.7, *p* < 0.001; Figures 2A,C).

### Sinomenine Prevented Myocardial Apoptosis in Myocardial Ischemia-Reperfusion Injury in Mice

**FIGURE 3:**
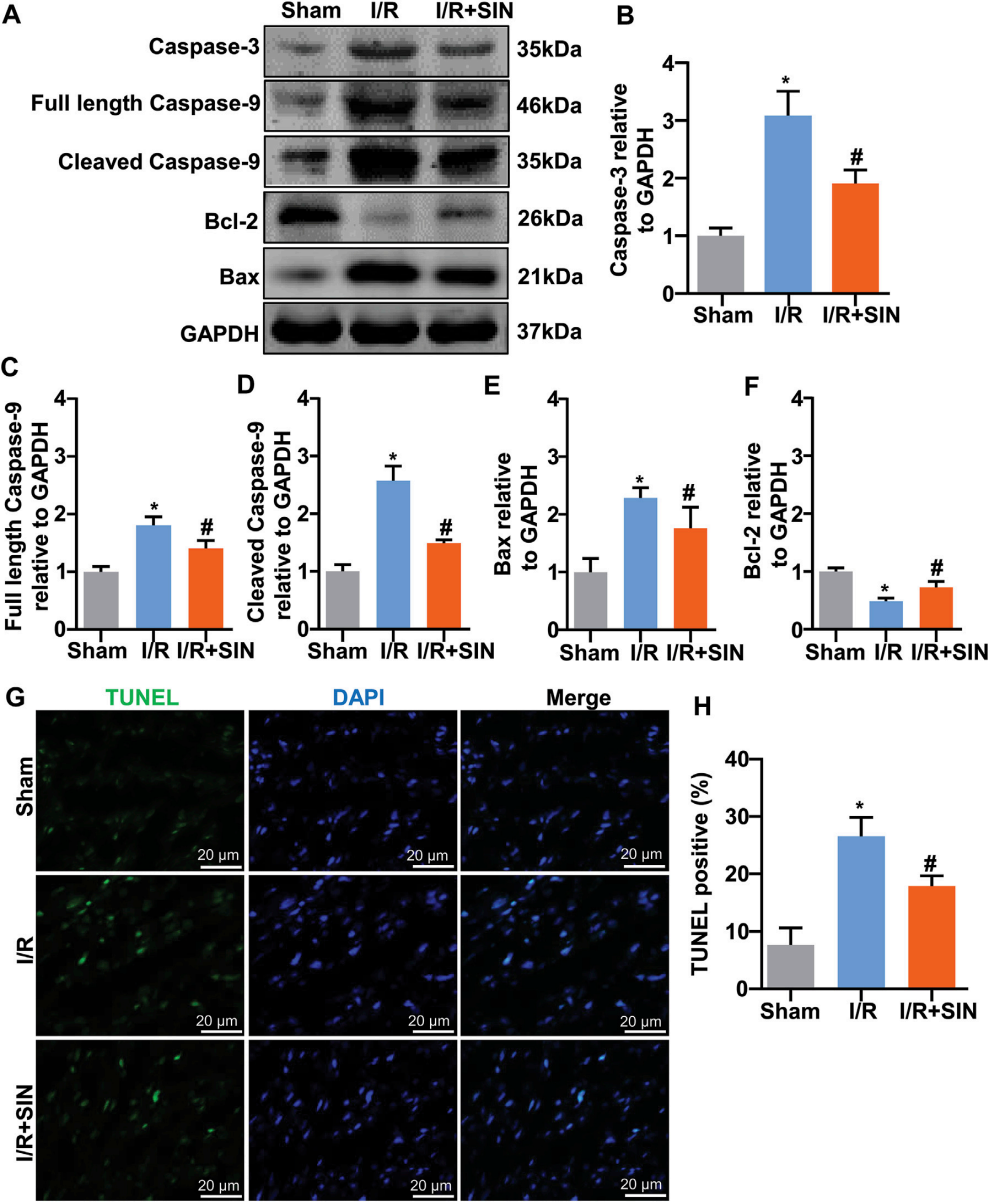
Effect of SIN on myocardial apoptosis induced by MIRI. **(A‒F)** Representative images and quantitative analysis show that pretreatment with SIN prevented the MIRI-induced increase in expression levels of caspase-3 **(A,B)**, full-length caspase-9 **(A,C)**, cleaved caspase-9 **(A,D)**, and Bax **(A,E)** in ischemic heart tissue and the MIRI-induced decrease in expression levels of Bcl-2 **(A,F)** (*n* = 5, *p < 0.05 vs. sham, ^#^ *p* < 0.05 vs. I/R). **(G,H)** Representative images **(G)** and quantitative analysis **(H)** show that pretreatment with SIN prevented the MIRI-induced increase in the number of TUNEL-positive cells in ischemic heart tissue (*n* = 5, *p < 0.05 vs. sham, ^#^ *p* < 0.05 vs. I/R). Data are shown as means ± SEM.

Next, we examined the effect of pretreatment with SIN on the apoptotic state of cardiac cells by detecting the changes in the expression of apoptosis-related proteins, such as caspase-3, caspase-9, Bax, and Bcl-2. The results showed that pretreatment with SIN significantly prevented the I/R-induced increase in the expression levels of caspase-3 in ischemic heart tissue (F_2, 12_ = 63.99, *p* < 0.001; Figures 3A,B). The pathologically increased expression levels of full-length (F_2, 12_ = 49.11, *p* < 0.001; Figures 3A,C) and cleaved caspase-9 (F_2, 12_ = 119.5, *p* < 0.001 Figures 3A,D) in ischemic heart tissue were also prevented by pretreatment with SIN. The expression levels of Bax (F_2, 12_ = 28.57, *p* < 0.001; Figures 3A,E) and Bcl-2 (F_2, 12_ = 56.69, *p* < 0.001; Figures 3A,F) were increased and decreased, respectively, in ischemic heart tissue, and these changes were significantly prevented by pretreatment with SIN. TUNEL assays showed that pretreatment with SIN remarkably prevented the I/R-induced increase in the number of TUNEL-positive cells in ischemic heart tissue (F_2, 12_ = 58.98, *p* < 0.001; Figures 3G,H).

### Sinomenine Prevented Oxidative Stress in the Myocardium of Myocardial Ischemia-Reperfusion Injury in Mice

**FIGURE 4:**
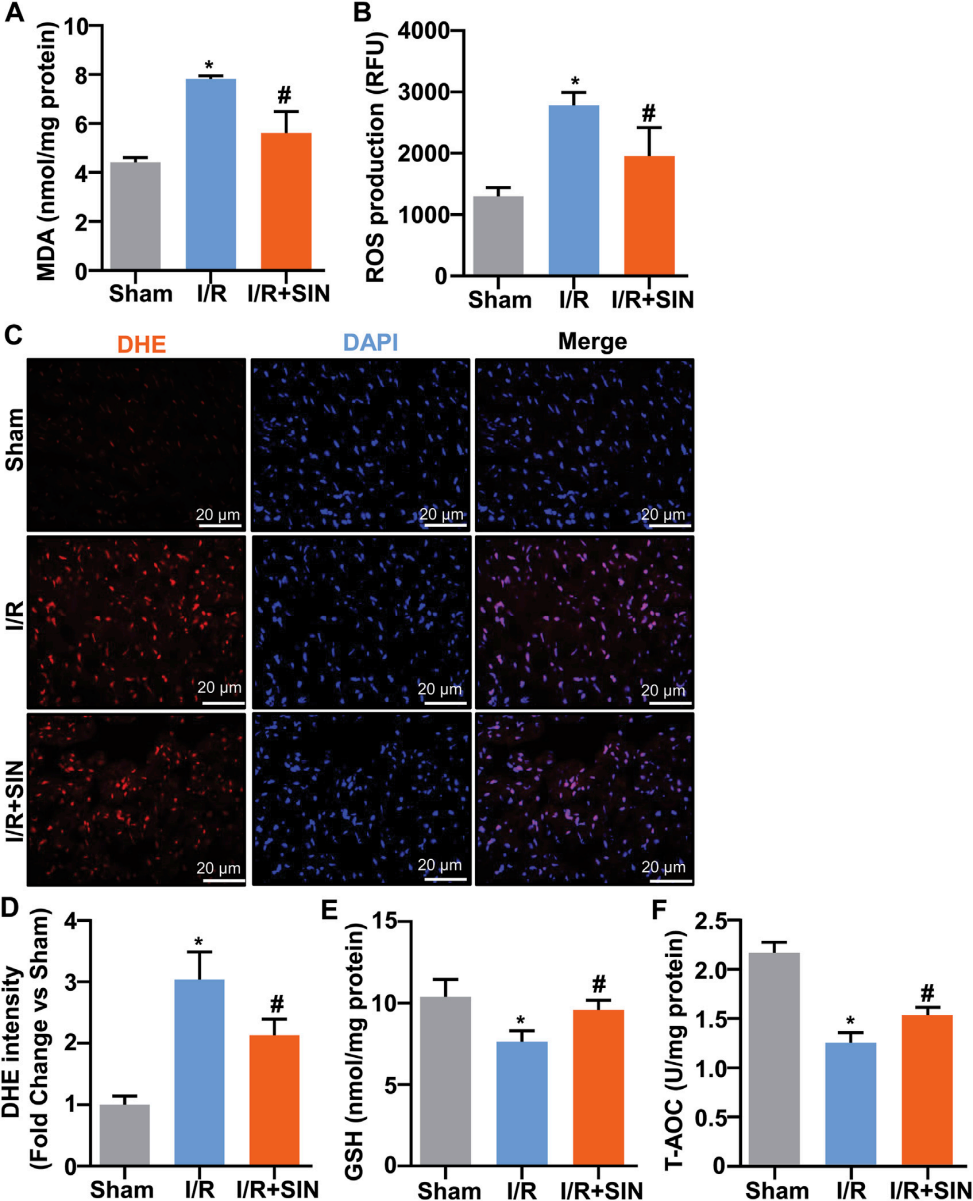
Effect of SIN on parameters reflecting oxidative stress in myocardial tissue of MIRI mice. **(A,B)** Quantitative analysis show that SIN pretreatment prevented the MIRI-induced increase in MDA (A, *n* = 4) and mitochondrial ROS (B, *n* = 6) levels in ischemic heart tissue (*p < 0.05 vs. sham, ^#^ *p* < 0.05 vs. I/R). **(C,D)** Quantitative analysis show that pretreatment with SIN prevented MIRI-induced increase in DHE signals in ischemic heart tissue (*n* = 5, *p < 0.05 vs. sham, ^#^ *p* < 0.05 vs. I/R). **(E,F)** Quantitative analysis showed that SIN pretreatment prevented the MIRI-induced decrease in GSH (*n* = 4) and T-AOC (*n* = 4) concentrations in ischemic heart tissue (*p < 0.05 vs. sham, ^#^ *p* < 0.05 vs. I/R). Data are shown as means ± SEM.

We next examined the changes in markers reflecting oxidative stress by determining the levels of GSH, T-AOC, and MDA in the cardiac tissue of mice treated with or without cardiac I/R or SIN. The results showed that pretreatment with SIN significantly prevented the cardiac I/R-induced increase in MDA (7.81 ± 0.07 with I/R vs. 5.61 ± 0.44 with I/R + SIN; F_2, 9_ = 43.29, *p* < 0.001; Figure 4A) and ROS (2779 ± 87.3 with I/R vs. 1958 ± 188.9 with I/R + SIN; F_2, 15_ = 35.36, *p* < 0.001; Figure 4B) in the ischemic heart tissue of mice. The results of DHE analysis were consistent with the ROS experiment, in which SIN treatment prevented the increase in DHE intensity in ischemic heart tissue induced by I/R treatment of the heart (3.04 ± 0.20 in I/R vs. 2.14 ± 0.11 in I/R + SIN; F_2, 12_ = 54.15, *p* < 0.001; Figures 4C,D). We also found that SIN pretreatment prevented the cardiac I/R-induced decrease in GSH (7.47 ± 0.32 in I/R vs. 9.88 ± 0.29 in I/R + SIN; F_2, 9_ = 21.21, *p* < 0.001; Figure 4E) and T-AOC (1.24 ± 0.05 in I/R vs. 1.52 ± 0.05 in I/R + SIN; F_2, 9_ = 83.83, *p* < 0.001; Figure 4F) in ischemic heart tissue. These results demonstrate that SIN can rebalance the pro- and anti-oxidative stress response in ischemic cardiac tissue.

### Sinomenine Prevented the Production of Pro-Inflammatory Cytokines in Myocardial Ischemia-Reperfusion Injury in Mice

**FIGURE 5:**
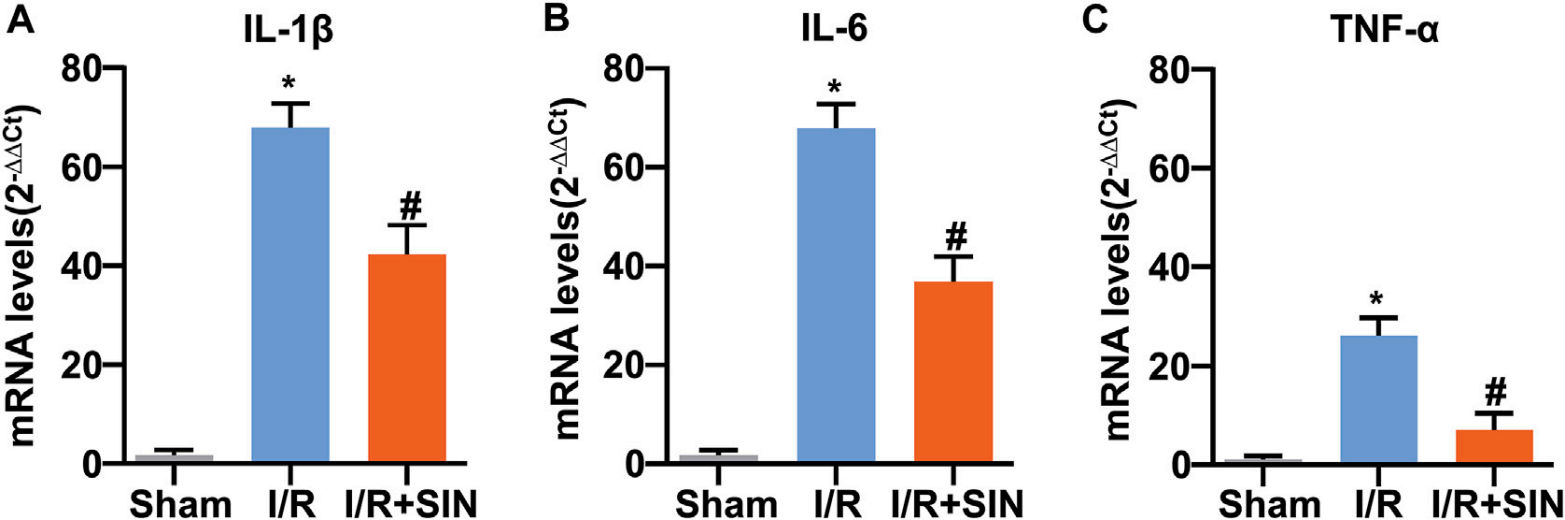
Effect of SIN on inflammatory parameters in myocardial tissue of I/R mice. **(A‒C)** Quantitative analysis show that pretreatment with SIN prevented the MIRI-induced increase in the expression levels of IL-1β **(A)**, IL-6 **(B)**, and TNF-α **(C)** mRNA in ischemic heart tissue (*n* = 8, *p < 0.05 vs. sham, ^#^ *p* < 0.05 vs. I/R). Data are shown as means ± SEM.

Finally, we examined the effects of SIN on the expression levels of IL-1β, IL-6, and TNF-α mRNA in cardiac tissue of mice treated with or without cardiac I/R. As shown in Figures 5A–C, the increase in the expression levels of IL-1β (F_2, 21_ = 445.00, *p* < 0.001, Figure 5A), IL-6 (F_2, 21_ = 522.80, *p* < 0.001, Figure 5B), and TNF-α (F_2, 21_ = 167.10, *p* < 0.001, Figure 5C) mRNA in the ischemic heart tissue of mice was partially but significantly prevented by pretreatment with SIN. This finding suggests that administration of SIN can prevent the progression of pro-inflammatory responses in ischemic heart tissue.

## Discussion

Since MIRI is the main cause of deterioration of cardiac function after coronary bypass or myocardial infarction, much attention has been paid to the search for effective drugs to treat this type of disease (Arslan et al., 2010). In previous work, SIN has shown *ex vivo* protection against arrhythmia, oxidative stress, and inflammation in a rat model of MIRI (Xie and Jin, 1993; Zhang et al., 2021; Lu et al., 2022). However, the isolated heart cannot fully represent the heart *in vivo* under normal physiological conditions because it is not regulated by the sympathetic nervous system, parasympathetic nervous system, and renin-angiotensin-aldosterone system (RAAS) (Papa, 2020; Martin et al., 2022). In addition, perfusion with Krebs-Henseleit buffer (KHB) instead of blood leads to the absence of physiological substances such as hormones, enzymes, and platelets (Papa, 2020; Martin et al., 2022). Moreover, hepatic drug metabolism is absent because SIN was administered in perfusate in the *ex vivo* model. Therefore, in the present study, we used the *in vivo* mouse model to investigate the effect of SIN in MIRI, which can better simulate the uptake and effect of SIN in the body and provide better evidence for clinical translation. We found that perioperative administration of SIN prevented MIRI-induced cardiac injury in mice, as initially evidenced by suppression of the MIRI-induced increase in serum levels of CK and LDH. During the pathological processes of MIRI, the elevated CK and LDH would leak from the cell membrane of the myocardium into the bloodstream and promote myocardial damage (King et al., 2010). Thus, by suppressing the pathological increase in serum levels of CK and LDH, SIN might be able to improve cardiac functions under the conditions of MIRI. This hypothesis could be supported by the following results: pre-treatment with SIN was found to prevent I/R-induced deterioration of cardiac function, as shown by the improvement of EF and FS and the decrease of infarct size in cardiac I/R mice.

**FIGURE 6:**
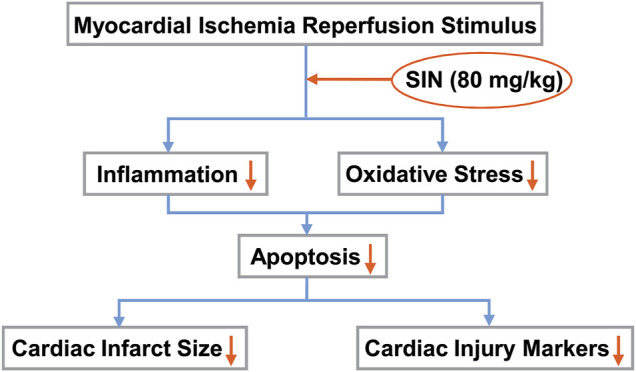
Schematic illustration showing that SIN provides protection against MIRI by preventing oxidative stress and inflammation. Pretreatment with SIN attenuated MIRI-induced inflammation and oxidative stress, thereby reducing cellular apoptosis and improving myocardial infarct size and markers of cardiac injury.

Oxygen is normally the driving force of numerous biochemical reactions that provide energy through oxidative phosphorylation. However, active metabolites of oxygen can also cause deleterious effects, of which oxidative stress is a well-known one. Oxidative stress is defined as an imbalance between the formation of ROS and antioxidant defenses. It is known to be functionally involved in the initiation of many pathological processes, such as myocardial damage, atherosclerosis, cancer, diabetes, and neurodegenerative diseases (Griendling and Alexander, 1997; Dröge, 2002). During ischemia-reperfusion of the myocardium, the mitochondria of the heart are both the major source of ROS formation and the site of the deleterious effects of ROS. The increased intracellular ROS eventually leads to oxidative stress and tissue damage. Therefore, strengthening antioxidant defenses may be beneficial for improving cardiac function. T-AOC is an antioxidant enzyme that can be depleted by the overproduction of ROS or lipid peroxidation of the membrane, which produces MDA. GSH is another intracellular antioxidant that serves as a substrate for anti-oxidative enzymes (Bułdak et al., 2014). High T-AOC and GSH levels or low MDA and ROS levels indicate a strong anti-oxidative capacity. Our results showed that pre-treatment with SIN could prevent the cardiac I/R-induced increase in MDA or ROS levels and the cardiac I/R-induced decrease in GSH and T-AOC levels in ischemic heart tissue. This highlights a possible role of SIN in attenuating oxidative stress-induced cardiac injury after MIRI stimulation (Figure 6).

In pathophysiological scenarios, a moderate increase in ROS levels can promote cell proliferation and survival, whereas massive ROS production can disrupt normal metabolism and induce cardiac apoptosis, a predominant consequence of MIRI (Kim et al., 2008; Das et al., 2014). The apoptotic process can be triggered by two pathways, intrinsic and extrinsic. The increased amount of ROS can initiate the mitochondria-mediated intrinsic apoptotic pathway by opening the mitochondrial permeability transition pore (Simon et al., 2000). Our TUNEL assay results showed that pretreatment with SIN prevented I/R-induced cellular apoptosis in ischemic cardiac tissue, which was consistent with the results for caspase-3 and caspase-9 detection (McIlwain et al., 2013). Meanwhile, we also examined the expression of Bcl-2 and Bax, two Bcl-2 family proteins that may regulate the intrinsic apoptotic pathway. Increased expression of Bcl-2 inhibits cell apoptosis, whereas increased levels of Bax promote apoptosis (Zhang and Saghatelian, 2013). The ischemia/reperfusion stimulus or oxidative stress acts as a stimulant to induce translocation of Bcl-2 family proteins such as Bax into mitochondria and alter their outer membrane to release pro-apoptotic proteins such as cytochrome C. The abnormally increased cytochrome C binds with the cytosolic protein apaf1 to form apoptosomes and activate the caspase-9 and -3 systems (Whelan et al., 2010). The upstream caspase-9 (initiator) activates the downstream caspase-3 (effector). Our results showed that SIN pretreatment resulted in a remarkable decrease in the expression levels of full-length caspase-9 and cleaved caspase-9 in ischemic heart tissue. We also observed a corresponding decrease in the expression of caspase-3 in ischemic heart tissue in mice pretreated with SIN. Analysis of Bcl-2 and Bax protein showed that pretreatment with SIN exerted an anti-apoptotic effect by preventing the expression of Bax and increasing the expression of Bcl-2 in ischemic heart tissue.

Mitochondrial ROS production may be related to mitophagy, another important target in the prevention of MIRI (Xia et al., 2021). In the process of mitophagy, damaged mitochondria are sequestered into the isolation membrane to form autophagosomes, fused with lysosomes, and finally removed from the cell (Li et al., 2021). Previous studies have demonstrated that the defective mitochondria in MIRI undergo adaptive mitophagy, which is cardioprotective to eliminate dysfunctional mitochondria, whereas excessive and dysregulated mitophagy disrupts cellular energy supply and exacerbates cell death (Kim et al., 2007; Li et al., 2021). Our work showed that pretreatment with SIN prevented mitochondrial accumulation of ROS. However, further research is needed to determine whether mitophagy is involved in the protection of SIN in MIRI and to investigate interactions between mitophagy adaptors.

The data from the real-time PCR experiment showed that pretreatment with SIN effectively suppressed the I/R-induced increase in the expression of pro-inflammatory cytokines (IL-1β, IL-6, and TNF-α) mRNA in ischemic heart tissue, suggesting that SIN may have an anti-inflammatory effect in I/R injury of the myocardium (Figure 6). The pathologically elevated pro-inflammatory cytokines could stimulate the biogenesis of ROS, leading to an enhanced inflammatory response, which subsequently promotes the recruitment of further inflammatory molecules into the injured myocardium in a positive feedback loop. Elimination of inflammation may reduce the oxidative stress burden, leading to a reduction in myocardial injury. We hypothesized that reducing ROS in cardiomyocytes by pretreatment with SIN might prevent the recruitment of inflammatory cells and thus the deleterious effects of inflammation on cardiac cells. This hypothesis should be investigated in future studies.

To date, antioxidant therapy appears to be disappointing in clinical trials because the mode of action of antioxidants is not understood (Forman and Zhang, 2021). For example, preventing free radical formation rather than scavenging ROS primarily protects cells from oxidative stress (Forman et al., 2014). Also, the dynamic interaction between antioxidant enzymes and their specific substrates can improve oxidative status (Forman and Zhang, 2021). Our results demonstrate the role of SIN in preventing the formation of ROS, which directly damages cardiomyocytes, increasing the activity of antioxidant enzymes, and inhibiting oxidant-induced inflammatory and apoptotic signaling. In addition, many researchers believed that antioxidants are susceptible to oxidation and therefore additional attention should be paid to the dose of the antioxidant for clinical use (Sharifi-Rad et al., 2020).

Future studies will investigate whether other signaling pathways are involved. One hypothesis is that the cardioprotective property of SIN may also be relevant to opioids. Opioids have been reported to have a cardioprotective effect against MIRI through the activation of opioid receptors (Schultz and Gross, 2001). Previous research suggests that SIN may mediate a nociceptive effect *via* μ-opioid receptors (Komatsu et al., 2019). Therefore, we speculate that SIN may exert an opioid-induced cardioprotective effect by inhibiting nociceptive signaling in the heart. We will therefore seek to perform a follow-up study to test this hypothesis.

One limitation is that there is no more than one reference gene. We used GAPDH as a reference gene, one of the most stable housekeeping genes in ischemic heart disease (Brattelid et al., 2010; Li et al., 2015). However, a range of reference genes is preferred for appropriate normalization in mouse cardiac research (Ruiz-Villalba et al., 2017). Another limitation is the single-dose administration of SIN. Although administration of 80 mg/kg SIN had the best protective effect in mice compared to lower doses and showed no observable side effects in mice according to multiple-dose studies of SIN, additional dose-response data and time-course data are needed to quantify the effects of SIN on the myocardial system (Gao et al., 2014; Zhu et al., 2014; Gao et al., 2018).

## Conclusion

A noteworthy conclusion from the above results is that pretreatment of SIN, by affecting oxidative stress and inflammatory pathways, is a promising way to treat I/R problems after infarction or surgery. The anti-inflammatory and antioxidant functions induced by SIN could attenuate cellular apoptosis and ultimately promote recovery from MIRI (Figure 6). Further in-depth studies are needed to uncover the molecular mechanisms by which SIN exerts antioxidant and anti-inflammatory protection of the heart. SIN, which targets oxidative stress and inflammation, could be a potential therapeutic strategy but remains to be tested in human studies.

## Data Availability

The original contributions presented in the study are included in the article/Supplementary Material, further inquiries can be directed to the corresponding authors.
